# Multi-Layered Hydrogels for Biomedical Applications

**DOI:** 10.3389/fchem.2018.00439

**Published:** 2018-09-25

**Authors:** Guiting Liu, Zhangfan Ding, Qijuan Yuan, Huixu Xie, Zhipeng Gu

**Affiliations:** ^1^Key Laboratory of Sensing Technology and Biomedical Instrument of Guangdong Province, School of Biomedical Engineering, Sun Yat-sen University, Guangzhou, China; ^2^State Key Laboratory of Oral Diseases, Department of Head and Neck Oncology, National Clinical Research Center for Oral Diseases, West China Hospital of Stomatology, Sichuan University, Chengdu, China

**Keywords:** multi-layered hydrogel, layer-by-layer self-assembly, step-wise, photo-polymerization, sequential electrospinning, biomedical application

## Abstract

Multi-layered hydrogels with organization of various functional layers have been the materials of choice for biomedical applications. This review summarized the recent progress of multi-layered hydrogels according to their preparation methods: layer-by-layer self-assembly technology, step-wise technique, photo-polymerization technique and sequential electrospinning technique. In addition, their morphology and biomedical applications were also introduced. At the end of this review, we discussed the current challenges to the development of multi-layered hydrogels and pointed out that 3D printing may provide a new platform for the design of multi-layered hydrogels and expand their applications in the biomedical field.

## Introduction

Hydrogels are three-dimensional polymeric networks cross-linked by physical, chemical interactions or a combination of both. Due to the hydrophilicity of the polymer chains in network, hydrogels can absorb and retain large amounts of water while immersed in aqueous solutions (Ahmed, 2015). A key property of hydrogel matrices is that they have not only viscoelastic properties like many soft tissues but also permeability to different types of molecules, such as proteins, bioactive agents and growth factors (Pan et al., 2011; Gupta et al., 2014; Lau and Kiick, 2015). Therefore, hydrogels have been frequently utilized in the field of drug delivery and tissue engineering (Hoffman, 2001; Highley et al., 2016; Fundueanu et al., 2017). In most instances, hydrogels are used as homogeneous soft materials with uniform bulk properties (Elisseeff, 2008). To better mimic anisotropic and complex structure of body tissues, hierarchical hydrogels containing multiple layers with different biochemical cues and various mechanical properties play crucial role for biomedical applications (O'Leary et al., 2011; Hu and Chen, 2014; Martínezsanz et al., 2016). Further, multi-layered hydrogels, formulated by the combination of multiple cell types, drugs or some other exogenous factors, could realize the controlled release behavior or cell interactions. With the emergence of biomaterials and fabricated technologies, the multi-layered hydrogels have been widely design for achieving adequate perfusion in biomedical applications (Christensen et al., 2018; Wang et al., 2018).

**Graphical Abstract:**
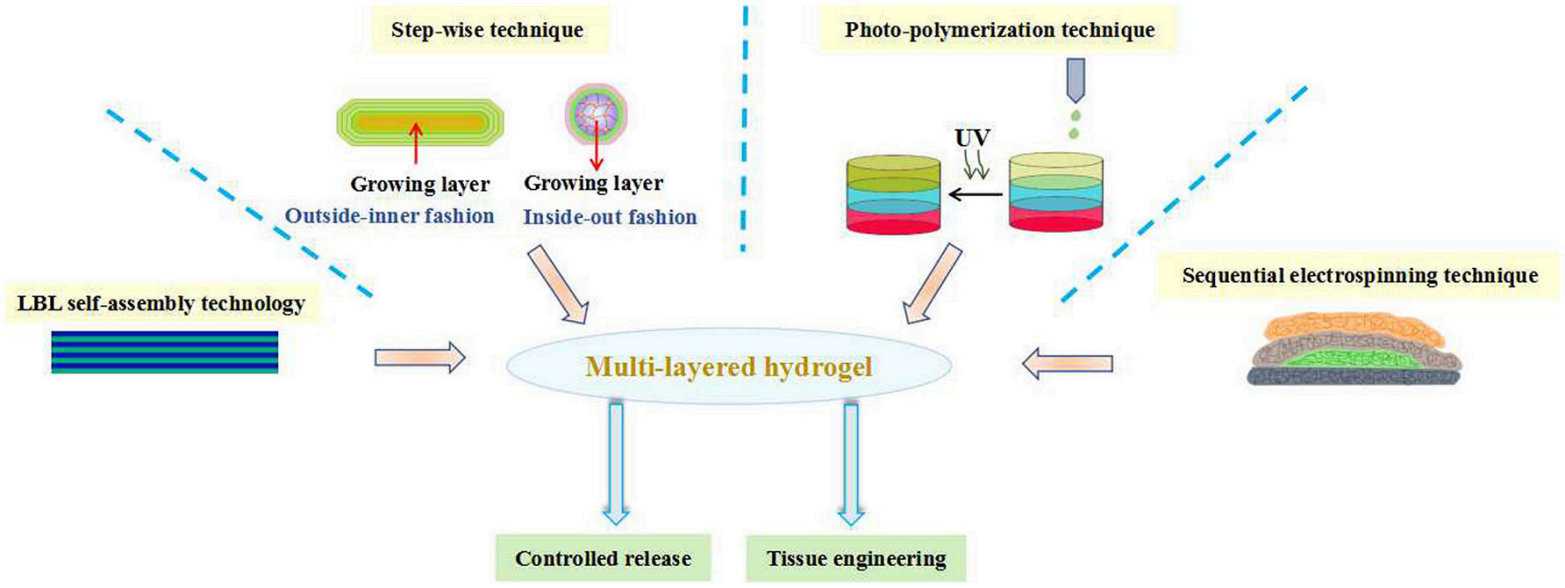
The preparation of multi-layered hydrogels and their biomedical applications.

In the past decade years, significant advances have been made in generating various multi-layered hydrogels. Most of these efforts so far have focused on engineering preparation of hydrogels with mimetic structure by different fabricating technologies. Since layer-by-layer (LBL) technology has attracted scientists to develop structure-controlled multi-layered hydrogels in the second half of the twentieth century, much important advancement has been brought in this field aim to ever closer achieving structural functions. In recent years, in addition to the development of multi-layered hydrogels by LBL technique, an increasing number of scientists are also becoming involved in step-wise technique, photo-polymerization technique, sequential electrospinning technique and 3D printing technique ect. Owing to their simplicity, versatility and robustness, multi-layered hydrogels could be manufactured as promising biomaterials for clinical applications. Until now, however, the overview, challenges and outlook of the multi-layered hydrogels in the field of biomedical applications have not been systematically reported. Herein, this review aims to summarize the recent progress in the fabrication of multi-layered hydrogels and their applications in biomedical fields. Further, the development of multi-layered hydrogels will be reviewed according to their preparation methods from four aspects and their morphology and biomedical applications will also be introduced. At the end, current challenges and future perspectives of multi-layered hydrogels for biomedical applications are mainly discussed.

## Multi-layered hydrogel based on layer-by-layer (LBL) self-assembly technology and its biomedical applications

At present, various technologies have emerged for the fabrication of multi-layered hydrogels. Among them, lay-by-layer (LBL) self-assembly technology has developed into the most commonly utilized method. In 1966, Iler et al. proposed the concept of LBL self-assembly for the first time. They used oppositely charged colloidal particles to alternately assemble on the glass substrate, and a film with controllable multi-layered structure was obtained (Iler, 1966). Since this pioneering work, LBL self-assembly technology has been extensively studied in the past few decades. The classic LBL self-assembly technology used the electrostatic interaction as a diving force to realize the process of assembly. With the deepening of study, a variety of driving forces have been developed to achieve LBL self-assembly, such as hydrogen bonding, covalent bonding, supramolecular complexation, biospecific recognition, charge transfer complex, etc. (Hoshi et al., 2002; Stuart et al., 2010; Xue et al., 2017). The development of LBL self-assembly technology provides a versatile tool for the preparation of multi-layered hydrogels, which generally possess ultrathin and alternating layered structure.

**Figure 1:**
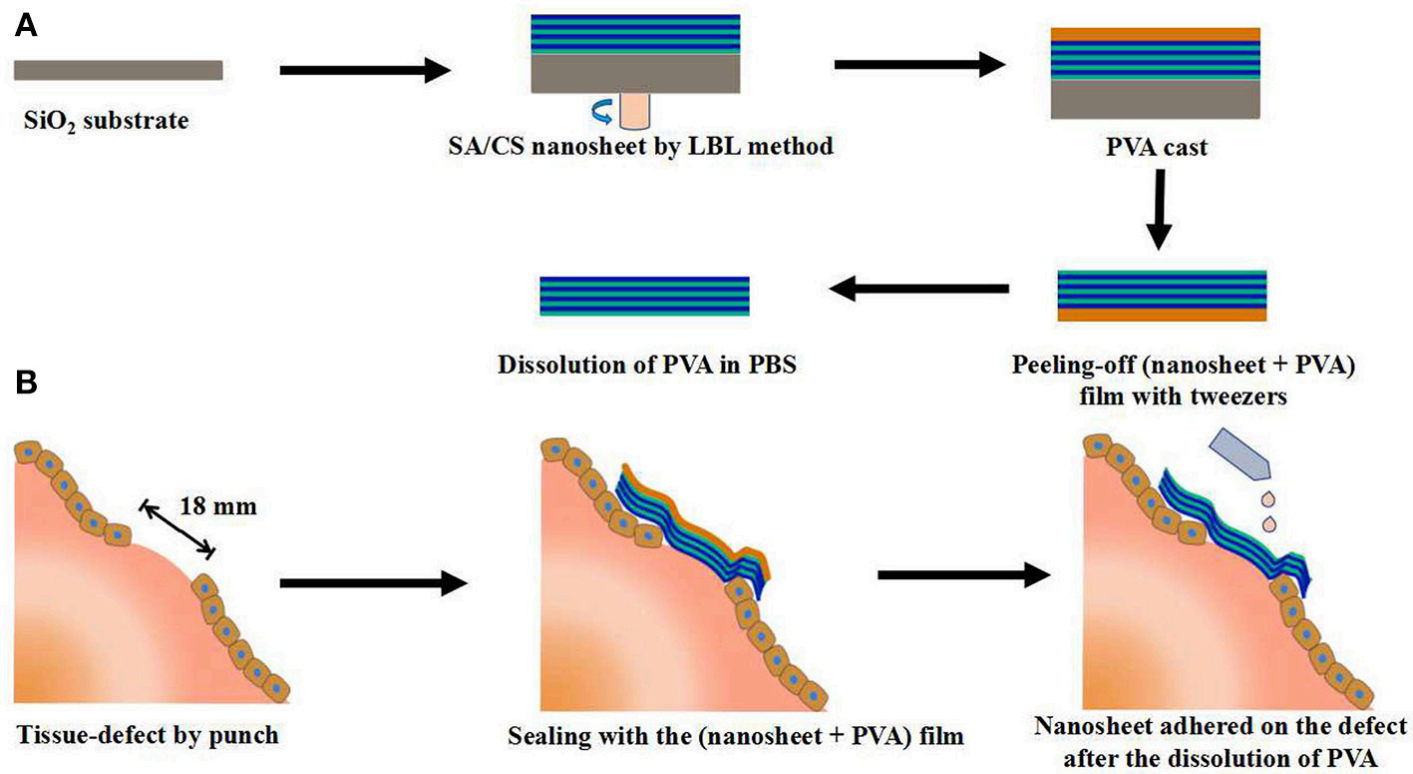
Schematic representation of the SA/CS nanosheet used for tissue-defect repair. **(A)** Fabrication of a free-standing SA/CS nanosheet by LBL method. **(B)** Schematic explanation of tissue-defect by SA/CS nanosheet.

Takeoka et al. prepared flexible, adhesive and robust polysaccharide nanosheet for surgical repair of tissue defects (Fujie et al., 2010). Alginate and chitosan, both of which are biocompatible and biodegradable, were selected as the building blocks to construct the nanosheet by spin-coating-assisted LBL method (Figure 1). The alternating layered structure of nanosheet was characterized by ellipsometric and IR spectroscopic test, which revealed that the thickness of one layer pair was about 2.9 nm, interfacial bonded by electrostatic interactions. The mechanical and physiological properties of the nanosheet could be easily modulated by the regulation of the concentration of polymers and the number of layers. The *in vivo* test showed that a nanosheet of 75 nm was suitable for the repairing of a visceral pleural defect in beagle dogs without any pleural adhesion. Such polysaccharide nanosheet has great potential as a novel tissue-defect repair material for various organs. In addition to the regulation of building blocks and the number of layers, the regulation of secondary structure could also contribute designable performance to the multi-layered hydrogels. For example, Hammond et al. prepared poly(ethylene oxide)-*block*-poly(ε-caprolactone) (PEO-*b*-PCL) micelles to carry the model drug triclosan. Then a multi-layered hydrogel film was fabricated by the alternating assembly of PEO-*b*-PCL micelles and poly(acrylic acid) (PAA) based on the hydrogen bond interactions in acidic solutions. Due to the weakness of hydrogen bonding, the multi-layered film disintegrated and released the PEO-*b*-PCL micelles under physiological conditions. Furthermore, the release period of triclosan could be extended by cross-linking the carboxylic acid groups of PAA layer (Kim et al., 2008). Li et al. prepared a glucose-sensitive multi-layered hydrogel film based on glucose oxidase (GOD) and insulin as well as a 21-arm star polymer by LBL self-assembly technology, driving by electrostatic interaction (Chen et al., 2011). The *in vitro* study indicated that such multi-layered hydrogel film could act as a responsive system switch to glucose, due to the pH-sensitivity of star polymer. Furthermore, this kind of multi-layered film had the potential to realize the multiple controlled release of insulin by the further modify of star polymer.

**Figure 2:**
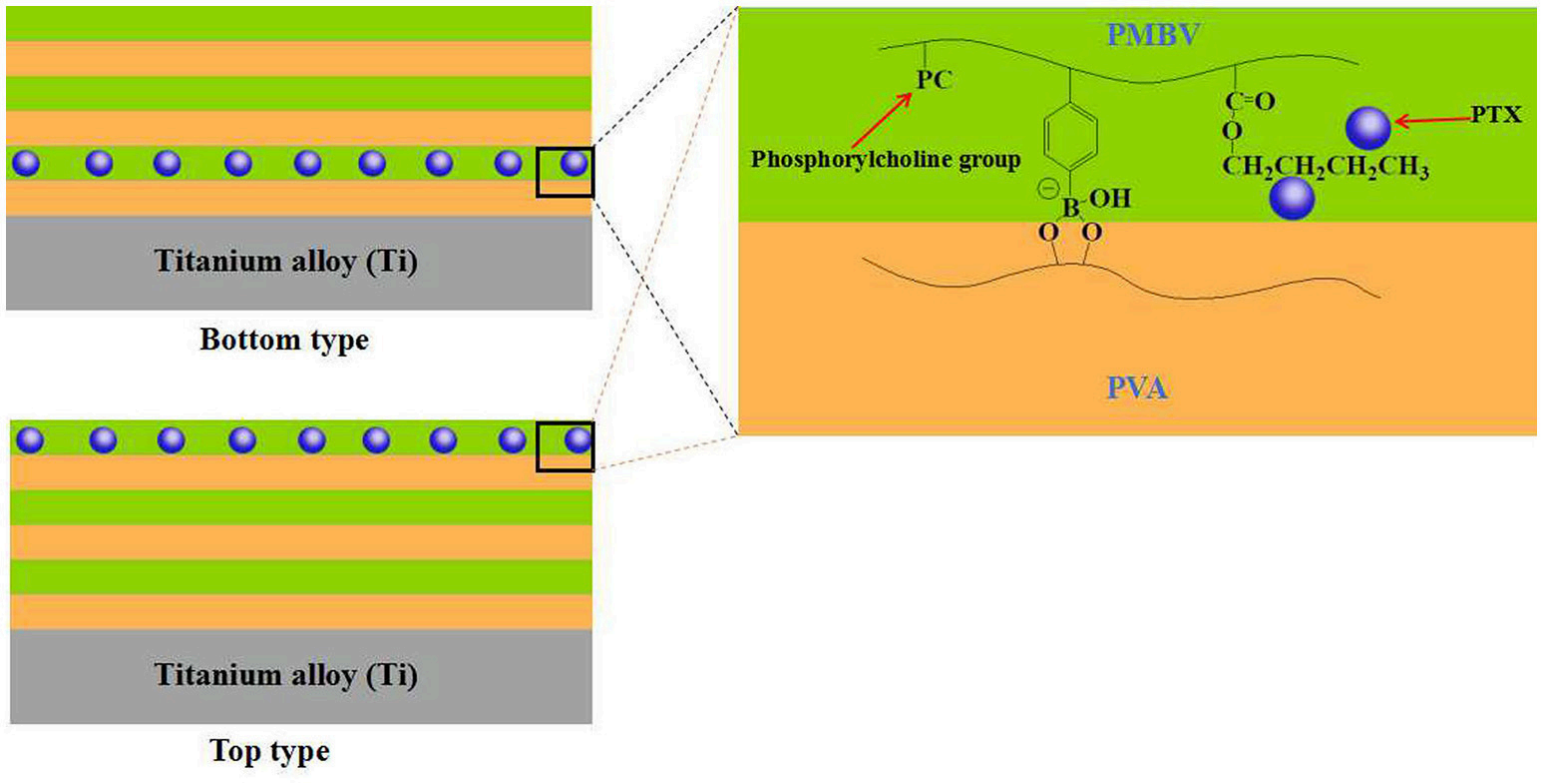
Schematic representation of PVA/PMBV multi-layered hydrogels with PTX incorporated in bottom and top layers.

In the past few decades, the application of implantable biomedical devices has achieved rapid development, such as artificial blood pump, intraocular lenses, artificial joint and cardiovascular stent (Moro et al., 2004; Mani et al., 2007; Guan et al., 2018). However, such applications face various problems, including metal hypersensitivity, infection and inflammation (Anderson, 2008; Lichter et al., 2016). In order to solve these problems, Ishihara's group did a lot of significantly work by the use of bioactive agents-loaded multi-layered hydrogel coatings on the implanted surface. For example, Ishihara et al. constructed a hydrogel coating with alternating six-layer structure on the surface of titanium alloy based on the synthesized phospholipid polymer (PMDV) and alginate by LBL technology, through the combination of electrostatic interaction and covalent bonding between PMDV and alginate (Choi et al., 2009b). Vascular endothelial growth factor (VEGF), a potent angiogenic signal transduction molecule, was selected as the model drug and loaded into the alginate layer by dispersing in alginate solutions in the process of self-assembly. The *in vitro* study showed that such multi-layered hydrogel coating achieved the sustained release of VEGF during one week. In another work, Ishihara et al. constructed a multi-layered hydrogel coating on titanium alloy surface based on another synthesized phospholipid polymer (PMBV) and poly(vinyl alcohol) (PVA) by LBL technology, driving by the reversible covalent bonding between boronic acid groups of PMBV and hydroxyl groups of PVA (Choi et al., 2009a). Paclitaxel (PTX), a poorly water-soluble anti-neoplastic agent, was dispersed in PMBV aqueous solutions due to the amphiphilicity of PMBV. During the layer stacking process of LBL technology, the PTX-loaded PMBV layer could be located on the designated layer of the multi-layered hydrogel coating. And the content of PTX increased with the number of PTX-loaded PMBV layer. The additional layers of PMBV and PVA, without PTX, could work as diffusion-barrier. In this way, the multi-layered hydrogel coating could enable the controlled release of PTX depending on the location and number of PTX-loaded PMBV layer (Figure 2). In a further work, Ishihara et al. studied the regulation of cellular proliferation by the PTX-loaded PMBV/PVA multi-layered hydrogels (Choi et al., 2012). The cell culture experiments showed that the cell proliferation of human epidermal carcinoma A431 cell could be controlled by the amount of released PTX. A desirable cell proliferation could be achieved by the PMBV/PVA multi-layered hydrogel with PTX loaded on an appropriate layer. These works suggested that the multi-layered hydrogel coatings fabricated by the LBL technology have the huge potential applications for the loading and surface-mediated delivery of bioactive agents from implantable biomedical devices.

As bacterial infection, especially related to the biomedical implants, is a great threat to patients, significant efforts have recently been focused on the design and fabrication of antibacterial materials (Pavlukhina and Sukhishvili, 2011; Busscher et al., 2012). Among these researches, the study of antibacterial hydrogel coating fabricated by LBL technology has attracted extensively attentions (Moskowitz et al., 2010; Xu et al., 2017). For example, Zhu et al. constructed ultrathin multi-layered hydrogel films based on the two synthesized PEG-based polymers POEGDMAM containing multi-enes and POEGMS containing multi-thiols by LBL technology, driving by the thio-ene “click” reaction (Wang et al., 2014). Owing to the presence of quaternary ammonium groups in the POEGDMAM, such multi-layered films exhibited significant antibacterial activity against both gram-positive and gram-negative bacteria, and the more layers the better the effect. Werner et al. fabricated bio-hybrid hydrogel coating on the surface of thermoplastic polyurethane substrates based on amino functionalized star-shaped PEG and EDC-s-NHS activated heparin (Fischer et al., 2015). Then silver nanoparticles were encapsulated into the star-PEG/heparin hydrogel when immersed into AgNO_3_ buffer solutions. Moreover, in order to regulate the release of silver ion, a silver-free star-PEG/heparin hydrogel was coated on the top of silver-loaded star-PEG/heparin hydrogel layer. The inhibition assay and blood incubation assay indicated that such multi-layered hydrogels could effectively combine antibacterial activity and hemocompatibility. In contrast to the traditional antibacterial hydrogel coating, Sukhishvili et al. fabricated bacteria-responsive hydrogel coatings without any antibacterial agents based on the self-defensive concept (Lu et al., 2015). They developed a family of polyanionic poly(2-alkylacrylic acids) (PaAAs) whose hydrophobicity could be modulated by their alkyl side chain length. Multi-layered hydrogels with varying hydrophobicity were constructed based on PaAAs and polyvinylpyrrolidone (PVPON) by LBL technology. The antibacterial activity assay indicated that when the medium turned acidic resulted from the bacterial proliferation, the PaAAs/PVPON hydrogels became more hydrophobic and dehydrated, and killed the bacterial contacted with the surface of hydrogel coatings. The more hydrophobic the PaAAs/PVPON hydrogels were, the better the killing efficiency was. Furthermore, the cell viability assays showed that such multi-layered hydrogel coatings exhibited no cytotoxicity to human osteoblasts.

## Multi-layered hydrogel based on step-wise technique and its biomedical applications

Nature inspired materials have attracted increasing attentions in the biomedical field for the past decades (Zhao et al., 2014; Zhang et al., 2016). Like onion, spinal disc and egg, all of which having many different layers organized in a concentric fashion, inspired the fabrication of onion-like multi-layered hydrogels. These onion-like hydrogels have great potential application in biomedical fields, such as cell bioreactors, drug delivery, evaluation of cell-cell interactions and tissue engineering (Kunze et al., 2011; Baek et al., 2013). More recently, the step-wise technique has developed into the most commonly utilized method for the construction of hydrogels with onion-like architectures in an inside-out or outside-inner fashion.

**Figure 3:**
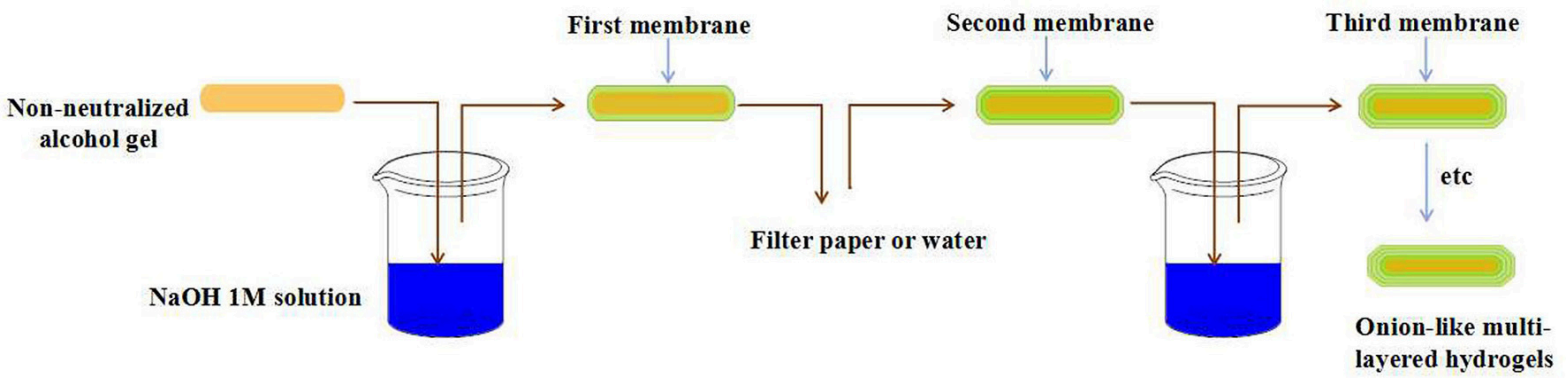
Schematic representation of chitosan multi-layered hydrogels prepared by step-wise technique.

Ladet et al. firstly reported the fabrication of onion-like hydrogels by controlling the kinetic gelation process of alginate or chitosan in an outside-inner fashion (Ladet et al., 2008). The precursor hydrogel could be easily prepared by evaporating water from the chitosan solution with water/1,2-propanediol mixed solvent. After the soaking of precursor hydrogel in NaOH and NaCl mixture solutions, the chitosan molecular chain was neutralized and physically cross-linked by hydrophobic interactions and hydrogen bonding due to the conversion of ${\text{NH}}_{3}^{+}$ to NH_2_. By controlling the kinetics of neutralization, the first membrane of precursor hydrogel could be formed. After the multi-step interrupted gelation process, the multi-layered chitosan hydrogel with onion-like architecture was obtained (Figure 3). By the same method, the onion-like alginate hydrogel was also prepared by the neutralization of precursor hydrogel in CaCl_2_ baths. In a further study, Ladet et al. investigated the bioactivity of the prepared onion-like chitosan hydrogel as chondrocytic cell bioreactors (Ladet et al., 2011). In this work, the articular rabbit chondrocytes were entrapped into the inter-membrane spaces of onion-like chitosan hydrogel and cultured for 45 days. This kind of multi-layered architecture had the ability to create the hypoxic physiological environment similar to the inter-vertebral disc tissues and cartilage. In addition, this multi-layered structure could prevent the cell penetration within the hydrogel matrix but allowed the protein diffusion throughout the hydrogel. The *in vitro* study revealed that chondrocytes not only formed cell aggregates and proliferated, but also produced numerous cartilage-type matrix proteins. Furthermore, no inflammatory markers were observed during the period of culture. Similarly, Hu et al. prepared multi-layer chitosan hydrogel by controlling the gelation process of chitosan solution, induced by acidic aqueous medium (Nie et al., 2015). Once the surface of chitosan solutions contacted with NaOH aqueous solutions, gelation started and the contact area transformed into a hydrogel layer instantly. Then the formed primary hydrogel acted as the medium between chitosan solutions and OH^−^ source. As the OH^−^ continued to diffuse, the onion-like chitosan hydrogel was formed with a layer-wise characteristic. By the utilization of such mechanism, multi-layered chitosan hydrogels with various architectures and shaped could be easily constructed in such an outside-inner fashion.

**Figure 4:**
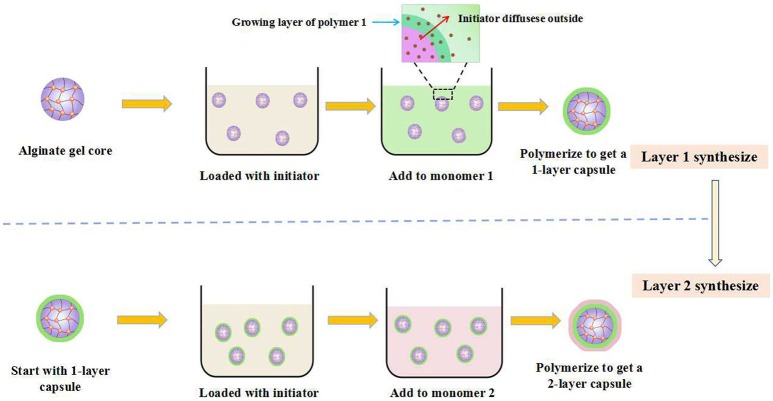
Schematic representation of onion-like hydrogels based on an inside-out fashion.

Besides the outside-inner fashion, the step-wise technique with an inside-out fashion, in which the precursor hydrogel core was constructed first and followed by the next different layers, was also commonly utilized to prepared multi-layer hydrogels with onion-like architecture. For example, Zhang et al. constructed onion-like cellulose hydrogel with high compressive strength and controllable architecture by a rapid contact of solid-liquid interface (He et al., 2014). Firstly, the agarose gel was prepared and worked as the precursor hydrogel core, and then soaked into acetic acid aqueous solutions. Subsequently, the agarose gel core with acetic acid was contacted with the cellulose solution with NaOH/urea mixture as solvent by a solid-liquid interface contact fashion. In this way, the first cellulose layer on the surface of agarose gel was formed, induced by the self-aggregation of cellulose chains resulted from the acid triggered destruction of cellulose inclusion complex in the NaOH/urea solutions. After the repeating of the above gelation process, the onion-like cellulose hydrogel was prepared. The architecture of the onion-like cellulose hydrogel, such as layer thickness, inter-membrane space and size, could be regulated by controlling the morphology of precursor hydrogel core and the time of solid-liquid interface contact time, as well as cellulose concentrations. The *in vitro* study showed that such cellulose hydrogel exhibited excellent biocompatibility and the L929 cells could adhere and proliferate in the inter-membrane space. This kind of biocompatible multi-layered cellulose hydrogel with controlled architecture has bright application prospects in biomedical application. Zarket and Raghavan developed a novel strategy to constructed onion-like hydrogels in an inside-out fashion (Zarket and Raghavan, 2017). Normally, a precursor hydrogel core within water-soluble initiator was prepared first, and then was immersed into a solution containing a monomer (monomer A) and a cross-linker. With the diffusion of initiator into the surrounding solution, the polymerization of monomer A was trigged and the first layer on the surface of precursor hydrogel core was formed. By repeating such immersion process in another solution containing monomer B and a cross-linker, the second layer was formed. In this way, various layers with controlled compositions and properties could be systematically constructed around the precursor hydrogel core (Figure 4). As a result, the mechanical properties and architectures of the prepared onion-like hydrogels could be easily regulated. These multi-layered hydrogels prepared by such strategy have great potential applications in the field of multi-stimuli responsive drug delivery.

## Multi-layered hydrogel based on photo-polymerization technique and its biomedical applications

Photo-polymerization is a process that utilizes light to initiate a polymerization reaction to obtain a linear or cross-linked polymer structure (Shi et al., 2016). Photo-polymerization technique has been frequently utilized to construct hydrogels, which were generally formed *in situ* with a non-invasive manner (Sudhakar et al., 2015; Brown et al., 2018). Recently, the photo-polymerization technique has developed into a powerful tool to prepare multi-layered hydrogels with complex architectures, which were generally used for tissue engineering.

**Figure 5:**
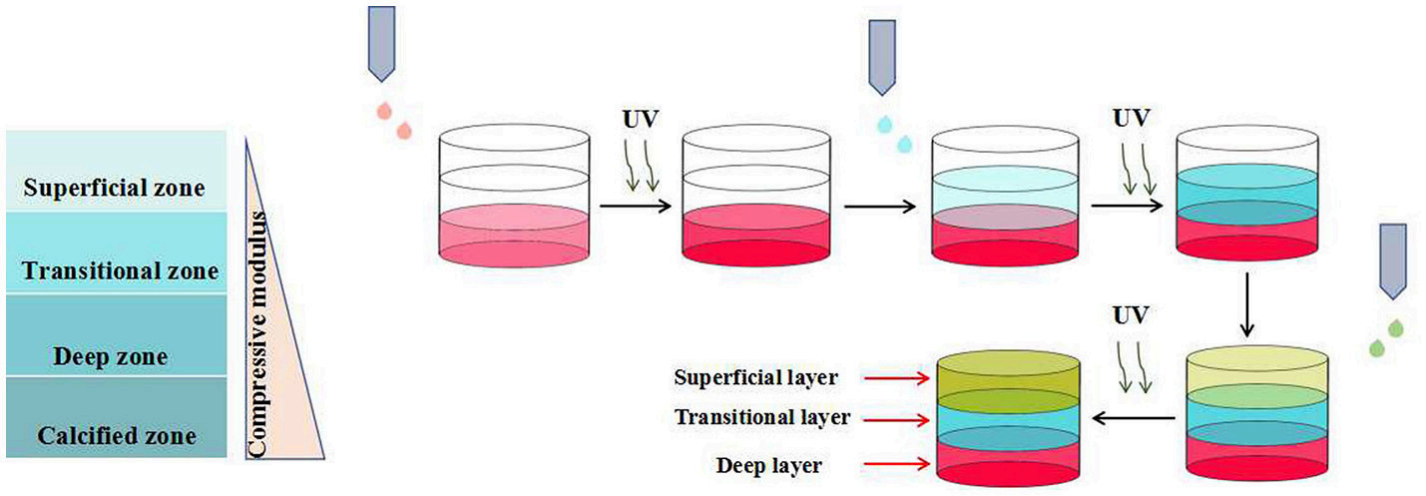
Schematic representation of three-layered hydrogels with spatially-varying mechanical properties by photo-polymerization technique.

Roy et al. prepared a novel kind of multi-layered hydrogel with spatially-varying mechanical properties and composition by photo-polymerization technique (Nguyen et al., 2011). Articular cartilage with spatially-varying structure is comprised of four zones: the superficial, transitional, deep, and calcified zones. In order to mimic the architecture and function of native articular cartilage, the authors prepared a multi-layered hydrogels with three distinctive layers, corresponding to the superficial, transitional and deep zones, respectively. The pre-designed each layer of the hydrogel, possessing gradual biochemical and mechanical properties, was assembled in a layer-by-layer fashion (Figure 5). Such strategy could also be applied to mimic other complex tissues with spatially-varying properties, such as artificial skin, joints and blood vessels. Similarly, Bryant et al. constructed a poly(ethylene glycol)-based hydrogel with spatially-varying biochemical and mechanical properties for osteochondral tissue engineering by sequential photo-polymerization (Steinmetz et al., 2015). Such hydrogel with three distinctive layers was fabricated to mimic the cartilage, calcified cartilage and bone zones of osteochondral tissues, respectively. The mechanical property of each layer was regulated by the concentration of macromer. And the biochemical property of each layer was modulated by varying the concentrations of chondroitin sulfate and RGD, both of which were selected as the extracellular matrix (ECM) molecules. Furthermore, the differentiation of human mesenchymal stem cells (hMSCs) within the multi-layered hydrogel matrix under intermittent dynamic compression was studied. The results indicated that dynamic mechanical stimulation could spatially guide hMSC differentiation efficiently. In another study, Bryant et al. constructed a multi-layered poly(ethylene glycol) (PEG) hydrogel with microchannels to promote the alignment of muscle cells (Hume et al., 2012). The inverse master mold with pre-designed width and depth was prepared by CLiPP polymerization first, then poly(ethylene glycol)diacrylate (PEGDA) solution was filled around the mold and irradiated by UV. After the removal of mold, PEG hydrogel with specific dimensions of macrochannels was formed. The *in vitro* study indicated that such three-dimensional long channels with width varying from 40 to 200 μm and depth varying from 100 to 200 μm promoted the muscle cell alignment significantly, and the smaller the channel dimensions was, the better the improvement was.

## Multi-layered hydrogel based on sequential electrospinning technique and its biomedical applications

Electrospun fibers have attracted numerous attentions due to their unique features, such as excellent mechanical properties, broad source of raw materials, high surface-area-to-volume ratio and designable morphology (Zucchelli et al., 2015; Wang and Windbergs, 2017). Sequential electrospinning technique, combining the advantages of electrospinning and multi-layering methodology, has also developed into a commonly used tool to construct multi-layered hydrogels for biomedical application (Kidoaki et al., 2005).

**Figure 6:**
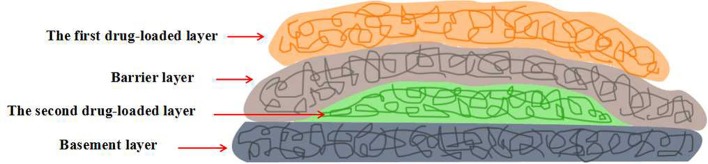
Schematic representation of four-layered hydrogels with time-programmed combination release behavior by sequential electrospinning technique.

Kidoaki et al. constructed a dual drug loaded multi-layered hydrogel with time-programmed combination release behavior by sequential electrospinning technique (Okuda et al., 2010). Four function layers were sequential fabricated: first drug-loaded layer, barrier layer, second drug-loaded layer and basement layer (Figure 6). The model drug was dissolved in electrospinning solutions and loaded *in situ* during the electrospinning process. The *in vitro* experiments indicated that the release behavior of each drug could be controlled by the fiber diameter and the layer thickness. Thereby, the combination release of the dual drugs could be achieved by the pre-designed architecture of the multi-layered hydrogels. Such strategy has bright future in the fabrication of multi-layered hydrogels for combination therapy application. Cho et al. prepared polycaprolactone (PCL) nanofibers reinforced alginate (SA) hydrogels by sequential electrospinning technique (Jang et al., 2013). Briefly, PCL electrospun fibers were treated with ethanol, improving cohesion with SA hydrogel matrix, and evenly distributed on SA/CaCO_3_ mixture solution, which was reapplied every five layers. With the repeating of such process, multi-layered SA hydrogels with various PCL nanofibers content were prepared. Compared with the pure SA hydrogels, the compressive strength and stiffness of PCL nanofibers reinforced SA hydrogels were improved by about 121 and 334%, respectively. In addition, the mechanical properties of such multi-layered composite hydrogels could be regulated by modulating the content of PCL nanofibers. Such mechanical property-enhanced hydrogels based on biocompatible polymer have great application as biomedical implants. Tuan et al. constructed a novel composite hydrogel scaffold based on fibrous poly-ε-caprolactone (PCL) and methacrylated gelatin (mGLT) for tendon tissue engineering by dual-electrospinning (Yang et al., 2016). After the co-electrospinning, composite scaffold could convert into composite hydrogel with uniform composition by photo-crosslinking retained mGLT. In this way, multi-layered composite hydrogels, mimicking native tendon tissues, could be fabricated by the photo-crosslinking of stacked scaffold sheets. Furthermore, cell seeding and photo-crosslinking could be performed simultaneously and the cell proliferation tests revealed that human ASCs impregnated into the multi-layered hydrogel still kept response to topographical cues and external tenogenic factors.

## 3D printing technique

**Figure 7:**
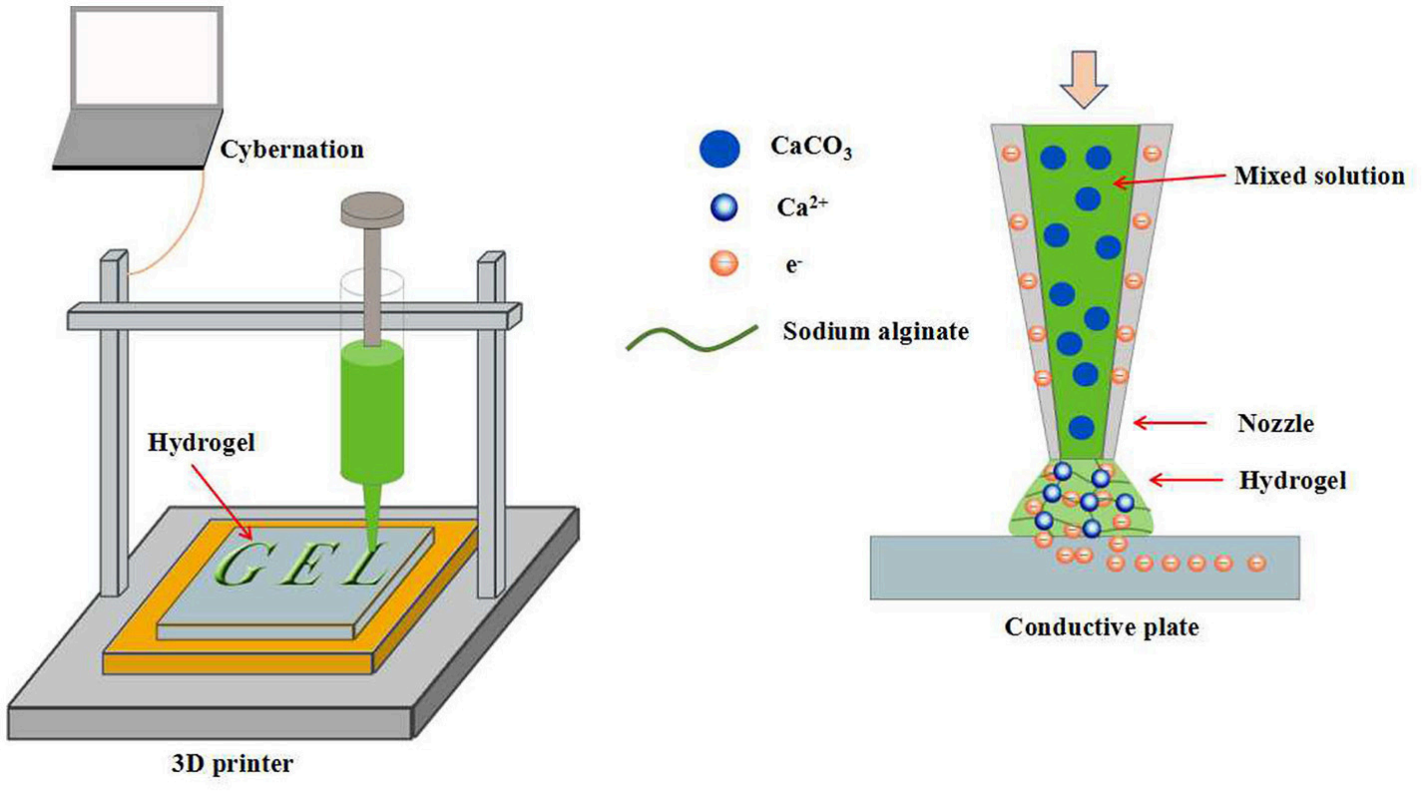
Schematic representation of calcium alginate hydrogels with controllable geometry by 3D printing.

3D printing is a kind of rapid prototyping technology. It is a technology based on a digital model file and uses a powdery metal or plastic adhesive material to build an object by layer-by-layer printing (Rengier et al., 2010; Sun et al., 2013; Chia and Wu, 2015). Recently, numerous attentions have been attracted by the hydrogels prepared by 3D printing for biomedical applications. For example, Shang et al. used a conventional 3D printer to fabricate 3D calcium alginate hydrogels (Shang et al., 2017). Sodium alginate and CaCO_3_ nanoparticles were premixed and filled into the syringe as 3D printing ink. Such mixture could be ejected onto a conductive substrate continuously by syringe nozzle and cross-linked by the Ca^2+^ released from the CaCO_3_ particles after the applying of a DC voltage between the substrate and the nozzle. A hydrogel formation model can be established by the consideration of several parameters, such as deposition time and applied voltage. The cell viability test revealed that the encapsulated cells can maintain 99% survive rate after the gelation. In addition, 3D printing can fabricate calcium alginate hydrogels with controllable geometry, which expanded their biomedical applications (Figure 7). The layer-by-layer printing characteristics of 3D printing inspired us to construct multi-layered hydrogels by the alternating printing of various materials. Preparation of multi-layered hydrogels by 3D printing has many advantages, such as controllable hydrogel morphology, engineering preparation, etc. However, similar work is rarely reported. Maybe in the near future, multi-layered hydrogels prepared by 3D printing will emerge in the field of biomedical applications.

## Conclusions and outlook

In the past decades, multi-layered hydrogels with organization of various functional layers have been attracting extensive attentions, due to their ability to meet the diverse needs of biomedical applications. Although the preparation of multi-layered hydrogels has made great progress, some challenges still remained. For example, LBL self-assembly technology, the most commonly used methods for the preparation of multi-layered hydrogel, faces the following problems: (i) The construction process is carried out in a layer-by-layer fashion and the operation is cumbersome, which is not conducive to the construction of hydrogels with numerous layers and the engineering preparation of multi-layer hydrogels. (ii) The thickness of each layer within multi-layered hydrogels cannot be adjusted flexibly. (iii) The range of drugs suited to such operation is narrow and the efficiency of drug loading is low. For the other remaining preparation methods, they not only face the similar challenges as LBL self-assembly technique, but also cannot achieve the fine control of architecture structure of multi-layered hydrogels. These problems limit the performance regulation and engineering preparation of multi-layered hydrogels, which seriously restricts their application prospects in the biomedical field. Therefore, in order to overcome the shortcomings of the existing preparation techniques and further increase the application potential of multi-layered hydrogels, it is urgently needed to develop a completely new methodology for the preparation of multi-layered hydrogels. More recently, 3D printing has developed into a versatile tool to create a three-dimensional object with almost any shape or geometry (Rengier et al., 2010; Ladd et al, 2013). Such process is rapid and intelligent, and is very suitable for the engineering preparation of objects with special structures. 3D printing was used to print metal powders in the early days, now it can print numerous polymer materials (Schubert et al., 2014; Ruminski et al., 2018; Tahayeri et al., 2018). Up to now, a large amount of literature has reported on the biomedical application of hydrogels generated by 3D printing (Bose et al., 2013; Gross et al., 2014; Muth et al., 2014). However, as far as we known, little literature reported the preparation of multi-layered hydrogels via 3D printing. Maybe in the near future, 3D printing can provide a new platform for the design of multi-layered hydrogels and expand their applications in the biomedical field.

## Author contributions

GL, ZD, and QY prepared the manuscript and figures. ZG and HX revised and finalized the manuscript. All authors reviewed and approved the final paper.

### Conflict of interest statement

The authors declare that the research was conducted in the absence of any commercial or financial relationships that could be construed as a potential conflict of interest.
